# Retrospective Analysis of the Effect of Lidocaine Combined with Methylprednisolone on Pain Control After Uterine Artery Embolization

**DOI:** 10.3389/fsurg.2022.875484

**Published:** 2022-04-19

**Authors:** Yi Tang, Bin Lin, Yan-ping Zhang, Ya-nan Hu, Jian-hui Zhang, Shao-jie Wu, Yan-feng Zhou, Sen-lin Cai, Jie-wei Luo, Wu Chi, Zhu-ting Fang

**Affiliations:** ^1^ Department of Shengli Clinical College, Shengli Clinical Medical College of Fujian Medical University, Fuzhou, China; ^2^ Department of Interventional Radiology, Fujian Provincial Hospital, Fuzhou, China; ^3^ Department of Traditional Chinese Medicine, Fujian Provincial Hospital, Fuzhou, China; ^4^ Emergency Department, Fujian Provincial Hospital, Fuzhou, China

**Keywords:** intra-arterial lidocaine, pain control, uterine artery embolization, retrospective analysis, methylprednisolone

## Abstract

**Background:**

The analgesic effect produced by the intra-arterial injection of lidocaine in patients undergoing uterine artery embolization has been proven to be safe and effective. Nevertheless, a significant degree of pain is typically experienced after the operation, and pain management is crucial. Methylprednisolone, which provides an anti-inflammatory effect, is widely used in the treatment of several diseases. To date, methylprednisolone has not been used after uterine artery embolization.

**Methods:**

A total of 131 patients with uterine leiomyoma were retrospectively enrolled. Forty-five patients (control group) were treated with embolized microspheres for bilateral uterine artery embolization. Fifty (study group) and 36 (lidocaine group) patients were administered lidocaine mixed with embolized microspheres during embolization, and in addition, the study group was administered methylprednisolone. Completed pain scales at different time points during surgery were obtained from patients undergoing uterine artery embolization. Efficacy against pain was evaluated by comparing the pain score, inflammatory index, and use of sufentanil within 24 h followed by a Kruskal-Wallis Test and a least significant difference post-hoc analysis.

**Results:**

The postoperative pain scores at 1, 4, and 7 h after uterine artery embolization in the study group (3.08 ± 2.09, 2.46 ± 1.93, and 2.38 ± 1.85, respectively) were significantly lower than those in the control group (4.84 ± 2.36, 4.16 ± 1.87, and 3.56 ± 1.93, respectively) and the lidocaine group (3.50 ± 2.10, 3.30 ± 1.88, and 3.28 ± 1.89, respectively). At the first 24 h after embolization, the total usage of sufentanil in the study group (31.4 ± 4.16) was significantly lower than those in the control group (45.7 ± 6.51) and the lidocaine group (38.3 ± 6.25). At 1 and 4 h, the pain scores of the lidocaine group were significantly lower than those of the control group. In addition, at the first 24 h after embolization, the total usage of sufentanil in the lidocaine group was significantly lower than that in the control group.

**Conclusion:**

Lidocaine in combination with methylprednisolone can significantly alleviate pain and reduce the usage of sufentanil after bilateral uterine artery embolization. Thus, methylprednisolone is a recommended addition to the therapeutic regimen after embolization.

## Introduction

Uterine fibroids are common tumors in women of childbearing age, causing symptoms such as increased menstruation and anemia (1). The most common treatments are drugs, surgery, and uterine artery embolization (UAE) (1). UAE can reduce uterine leiomyoma by 50%–60%, uterine size by 40%–50%, symptoms by 88%–92%, and abnormal uterine bleeding by more than 90%. It is a safe, effective, and minimally invasive method for the treatment of symptomatic uterine leiomyoma that has received extensive attention and has been used clinically for nearly 20 years (2). Compared with laparoscopic myomectomy, patients undergoing UAE experience a shorter hospital stay and faster recovery (3). Hysterectomy may be considered for women with large uterine fibroids who do not intend to become pregnant, and patients with fibroids greater than 10 cm maximum in diameter are more likely to be converted to laparotomy during laparoscopic hysterectomy (4). Therefore, UAE treatment can also be considered to help patients retain the uterus. However, postoperative pain usually begins 1 h after UAE and worsens within the next 5–7 h. Some patients experience difficulty in controlling the pain, but it usually subsides in the first 24 h after UAE (5). Hypogastric nerve block can relieve or eliminate ischemic pain caused by embolization; however, it is an invasive surgery (6). Recent studies have demonstrated a significant correlation between postoperative pain and myometrial ischemia, but not with the degree of embolization of the leiomyoma, volume of the uterus, or size of the leiomyoma (7). This condition is related to postembolization syndrome (PES) caused by necrosis and ischemic injury of normal tissue after UAE, which can provoke a systemic inflammatory response (8). Moreover, this response has also been confirmed in hepatic and splenic artery embolization (9).

Lidocaine is a widely used amide local anesthetic for pain relief caused by transcatheter arterial chemoembolization and peripheral arteriography (10, 11). However, in a study by Katsumori et al., the postoperative addition of lidocaine was not found to have a significant effect on pain improvement (12).

Methylprednisolone is an intermediate-acting glucocorticoid, and because of its anti-inflammatory effect, it is widely used in the treatment of several diseases (13).

At present, the efficacy of lidocaine in pain relief after UAE is controversial; therefore, we conducted this study to determine whether intravenous administration of methylprednisolone with a mixture of microspheres and lidocaine can reduce pain after UAE.

## Materials and Methods

### Patient Information

A total of 131 patients were diagnosed with uterine leiomyoma from December 2016 to December 2020, of whom 45 (control group) were treated with embolized microspheres for bilateral UAE from December 2016 to August 2018. From September 2018 to December 2020, 50 patients (study group) and 36 patients (lidocaine group), were administered embolic microspheres supplemented with 100 mg lidocaine in a volume of 20 mL, slowly injected into each uterine artery using a 1-mL syringe during the surgery. In addition, the study group was transferred back to the ward and received 40 mg of methylprednisolone as an intravenous infusion. All procedures were performed in accordance with the tenets of the Declaration of Helsinki, and the study was approved by the Ethics Committee of Fujian Provincial Hospital, Fuzhou, China. All participants and legal guardians of minors involved in the present study provided written informed consent.

The selection criteria (14) were as follows: (1) uterine leiomyoma confirmed by plain pelvic magnetic resonance imaging (MRI) and contrast-enhanced imaging within 1 week before surgery; (2) one or more symptoms such as abnormal uterine bleeding, dysmenorrhea, pelvic compression symptoms, or frequent/urgent urination; and (3) refusal of surgical treatment, immunocompromised status, or severe comorbidities precluding surgery.

The exclusion criteria (15) were as follows: (1) history of UAE or untreated leiomyoma with complete infarction; (2) history of allergy or intolerance to lidocaine or other amide anesthetics, methylprednisolone, or other glucocorticoid drugs; (3) subserosal or submucosal pedicled uterine leiomyoma, with a connecting section width less than 50% of the diameter of the myoma; (4) age >50 or <50 years and ongoing menopause; (5) acute or chronic pelvic (uterine, appendages, and urinary system) inflammation, intrauterine live birth, or uterine or ovarian pelvic organ malignant tumors; (6) chronic renal failure (glomerular filtration rate <60 mL/min, serum creatinine level >133 µmol/L); (7) coagulation disorders; (8) allergy to iodine-containing contrast media or contraindications to MRI examination; and (9) endometriosis or adenomyosis.

### UAE Treatment Process

#### Uterine Arteriography

UAE was performed on all patients by an experienced interventional radiologist (12 years of UAE surgery experience). The bilateral groin area was disinfected routinely under local anesthesia. The Seldinger technique was used to puncture the right or left femoral artery, and a 5F Cobra catheter (Cook, Inc., Bloomington, IN, USA) or hepatic artery catheter (Terumo, Tokyo, Japan) was used for angiography of the left and right internal iliac arteries at an ipsilateral 45° oblique position. The contrast media flow rate was 4 mL/s, with a total of 12 mL, and the injection pressure was 300 psi (1 psi = 6.895 kPa). Based on the results of the internal iliac angiography to identify the uterine artery, patients with severe vascular tortuosity or on whom superselective catheterization was difficult to perform could be intubated through the femoral artery on the other side. Under the guidance of the road map, the 2.6 FRAPIDTHRUTM mini-catheter guide wire system (Jiangsu Hengrui Disheng Medical Co., Suzhou, China) was superselectively inserted into the horizontal segment of the uterine artery and over the beginning of the cervicovaginal branch. Using orthotopic uterine arteriography, the contrast media flow rate was set to 2 mL, total volume was 6 mL, and injection pressure was 300 psi.

#### Uterine Artery Embolization

After confirmation of the uterine arteries, the bilateral uterine arteries were injected with 300–500 µm or 500–700 µm uterine fibroid bland embolization with PVA particles (Suzhou Hengrui Jialisheng) for embolization. The embolization microspheres were supplemented with 100 mg lidocaine in a volume of 20 mL, and in the study group and lidocaine group, the suspension was slowly injected into the uterine artery using a 1-mL syringe. Uterine artery embolic particles were injected slowly to avoid reflux and false embolisms. When the contrast medium was filled to the proximal end of the uterine artery, it remained for 5–10 min so that the embolic particles were redistributed and filled the blood vessels. The injection of the medium was then continued until it was retained in the blood vessels and the uterine artery cast appeared, indicating that embolization was complete. The microcatheter was withdrawn, and angiography was performed again to evaluate the degree of embolization and determine whether other collateral blood vessels were apparent. After removal of the catheter and sheath, puncture site hemostasis was achieved with manual compression. After the surgery, patients were returned to the ward, and 40 mg of methylprednisolone was administered intravenously to the study group patients within 1 h after the surgery. The right lower limb was immobilized for 24 h. After UAE, the patients were hospitalized for observation for 1–3 days, and the postoperative reactions and occurrence of complications were observed and recorded in detail.

### Efficacy Evaluation

#### Laboratory Examination of Serum Inflammation Indicators

One day before and after the surgery, the Beckman Coulter LH 750 automatic blood cell analyzer (Beckman Coulter, Brea, CA, USA) was used to detect white blood cell levels which were stratified into five classifications. The plasma procalcitonin level was determined by electrochemiluminescence, and the plasma C-reactive protein level was measured by immunoturbidimetry.

#### Pain Score

Pain assessment forms completed during the different time periods of the surgery were collected and analyzed. The assessment form is a routine assessment tool for all UAE patients during post-hospital treatment. The pain rating scale is based on the numerical rating scale (16), which can systematically evaluate the change in pain intensity after UAE surgery. The scoring scale uses numbers from 0 to 10 to indicate the degree of pain. A straight line was divided into 10 segments, and the degree of pain was evaluated from 0 to 10 by encircling the number indicating the degree of pain: 0, painless; 1–3, mild pain (pain does not affect sleep); 4–6, moderate pain; 7–9, severe pain (unable to fall asleep or wake up painfully during sleep); and 10, sharp pain.

#### Postoperative Pain Evaluation and Intervention Treatment

After the patient was returned to the ward, the nurse used a digital pain score for the patient hourly and performed pain intervention treatment based on the resulting score (17): ≤3 points, mild pain only, requiring clinical observation and no medication; ≥4 points, moderate pain, patient given sufentanil self-controlled intravenous analgesia pump (the dose used was 2.0 µg/kg of sufentanil and diluted to 100 mL with normal saline. The first dose was 2 mL, and the subsequent dose was 1.5 mL/h. If the pain still does not decrease after administration, the administration could be continued, the dose was 2 mL/time, and the pump should be completed within 15 min); and ≥7 points, severe pain, patient was administered remedial sufentanil as a single dose of 2 mL. The results of the digital pain score, analgesic drugs, and their dosages were recorded in detail in the patient medical records. The main endpoints of the study were the pain scores of patients during UAE surgery at 1, 4, 7, and 24 h after the surgery and the total amount of sufentanil administered 24 h after the UAE surgery.

#### Imaging Examination

Pelvic MRI scan + enhancement was performed within 1 week before the surgery to confirm the diagnosis of uterine leiomyoma and evaluate the size of the uterus and leiomyoma. Three months after the surgery, a review MRI was performed to assess the volume of the uterus and leiomyoma after surgery and the embolic degree of leiomyoma, so as to further determine the change in uterine and leiomyoma volume.

### Statistical Analysis

Statistical analyses were performed using SPSS analysis (SPSS Statistics for Windows, Version 22.0, IBM Corp., Armonk, NY, USA). The measurement data conforming to the normal distribution are expressed as mean ± standard deviation (SD$\bar{x}\;\pm \;s$). One-way analysis of variance (ANOVA) was applied, followed by Duncan test inspection verification and least significant difference post-hoc analysis. The measurement data that did not conform to the normal distribution were expressed by the median (M) and 25%–75% quartiles, and the differences were compared using the Kruskal–Wallis test. Chi-square test to compare count data among groups. A *p* < 0.05 was considered statistically significant.

## Results

### Clinical Efficacy Evaluation

**Figure 1:**
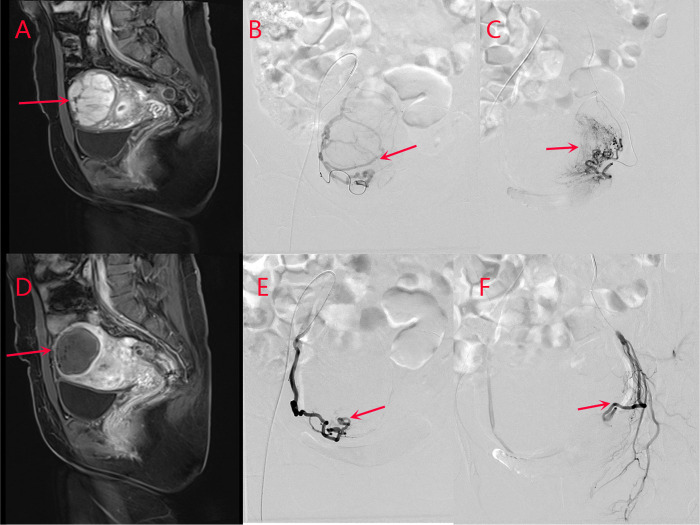
Images from a patient in the study group. (**A**) Before uterine artery embolization, and (**D**) after uterine artery embolization. The volume of uterine leiomyoma is reduced from 7.2 cm × 7.4 cm × 9.6 cm to 5.4 cm × 5.7 cm × 7.5 cm as revealed by pelvic MRI contrast, and the enhancement has disappeared. (**B**) Right uterine arteriography and (**C**) left uterine arteriography before uterine artery embolization. (**E**) Right uterine arteriography and (**F**) left uterine arteriography after uterine artery embolization. The bilateral leiomyoma arteries are basically occluded, and blood perfusion has disappeared completely after uterine artery embolization, suggesting the complete infarction of the myoma after uterine artery embolization.

**Figure 2:**
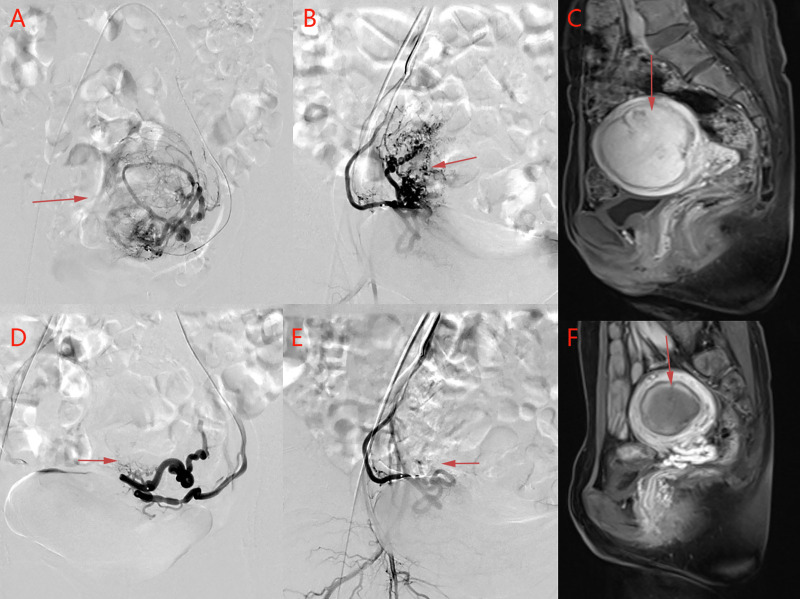
An image from a patient in the control group. (**A**) Left uterine arteriography and (**B**) right uterine arteriography before uterine artery embolization. (**D**) Left uterine arteriography and (**E**) right uterine arteriography after uterine artery embolization. The bilateral leiomyoma arteries are basically occluded after embolization. (**C**) Before uterine artery embolization and (**F**) after uterine artery embolization. Pelvic MRI contrasts show that the volume of uterine leiomyoma has decreased from 5.1 cm × 5.3 cm × 7.9 cm to 3.3 cm × 4.5 cm × 6.1 cm, and the enhancement has disappeared, suggesting that the myoma is completely infarcted after uterine artery embolization.

All patients underwent UAE successfully, and there were no significant differences in baseline data among the three groups (*p* > 0.05) (**Table 1**). There were no statistically significant differences in inflammatory indices among the three groups before embolization and 24 h after embolization (*p* > 0.05) (**Table 2**). MRI re-examination was performed 3 months after embolization and revealed that the uterine volumes and sizes of the leiomyomas in the three groups were significantly smaller than those before the embolization. The uterine volumes were reduced by approximately 53%, and there were no significant differences in the degree of change of the uterus and leiomyoma among the three groups (*p* > 0.05) (**Table 3** and **Figures 1A,D, 2C,F**).

**Table 1:** Comparison of baseline characteristics of clinical data between control group and study group in patients with uterine artery embolism.

Characteristic	control group (*n* = 45)	Lidocaine group(*n* = 36)	study group (*n* = 50)	F value or χ^2^ value	*P* Value
Weight, kg	57.2 ± 5.1	56.3 ± 5.3	55.8 ± 4.7	1.01	0.36
Body mass index	23.2 ± 3.8	22.4 ± 2.6	22.8 ± 2.9	0.66	0.52
Mean age (y)	45.4 ± 3.2	44.2 ± 3.6	46.7 ± 4.8	1.87	0.16
Mean uterine size (mL)					
Median	268.3	562.5	293.9	0.03	0.99
Range (25%–75%)	201.0–1095.6	258.3–953.1	195.5–1145.3		
Mean largest tumor size (mL)					
Median	67.3	163.8	99.8	0.49	0.78
Range (25%–75%)	51.6–258.6	103.3–331.5	56.8–503.4		
Primary symptom					
Menorrhagia (*n*)	36	31	41	0.53^a^	0.77
Dysmenorrhea (*n*)	20	23	26	3.05^a^	0.22
Pelvic pressure (*n*)	24	20	25	0.38^a^	0.83
Bloating (*n*)	39	35	43	3.27^a^	0.20
Constant urination (*n*)	31	30	38	2.27^a^	0.32

*Note: n: numbers.*

**Table 2:** Comparison of inflammatory indexes among three groups before embolization and 24 h after embolization.

Characteristic	pre-UAE			F Value	*P* Value	Pro-UAE			F Value	*P* Value
	Control group	Lidocaine group	Study group			Control group	Lidocaine group	Study group		
leukocyte (×10^9^/L)	7.1 ± 2.2	6.9 ± 1.9	6.5 ± 2.1	1.04	0.36	8.8 ± 3.1	8.3 ± 3.1	8.4 ± 3.5	0.54	0.58
Procalcitonin (ng/mL)	0.08 ± 0.03	0.08 ± 0.04	0.09 ± 0.03	1.61	0.20	0.09 ± 0.04	0.09 ± 0.04	0.10 ± 0.04	0.81	0.44
C reactive protein (mg/L)	5.04 ± 7.56	4.09 ± 4.83	4.51 ± 3.89	0.36	0.70	6.62 ± 7.02	5.44 ± 5.56	4.93 ± 7.48	0.96	0.39

Note: C reactive protein normal range,0–3 mg/L; Procalcitonin normal range,0–0.25 ng/mL; leukocyte normal range,3.5–9.5*10^9^/L. UAE, uterine artery embolization.

**Table 3:** Comparison of curative effects of three groups 3 months after operation by MRI evaluation in uterine artery embolization patients with Median (25%–75%).

Characteristic	3 months after operation			F Value	*P* Value	Reduction degree			F Value	*P* Value
	control group	Lidocaine group	study group			control group	Lidocaine group	study group		
Size of the uterus (mL)	165.4 (113.7–700.7)	345.7 (131.5–656.5)	177.9 (120.1–733.4)	0.02	0.98	105.7 (74.9–407.3)	223.9 (112.7–287.4)	117.1 (66.6–411.5)	0.10	0.90
Volume of hysteromyoma (mL)	30.9 (25.5–126.6)	65.1 (28.1–150.4)	44.1 (26.2–234.8)	0.40	0.96	38.4 (29.0–138.1)	104.5 (68.0–147.6)	55.9 (32.8–256.3)	0.11	0.90

*Note: MRI: Magnetic Resonance Imaging; Reduction degree = size of the uterus (mL) before embolization /size of the uterus (mL) after embolization.*

### Comparison of Uterine Artery Spasm During Surgery

**Figure 3:**
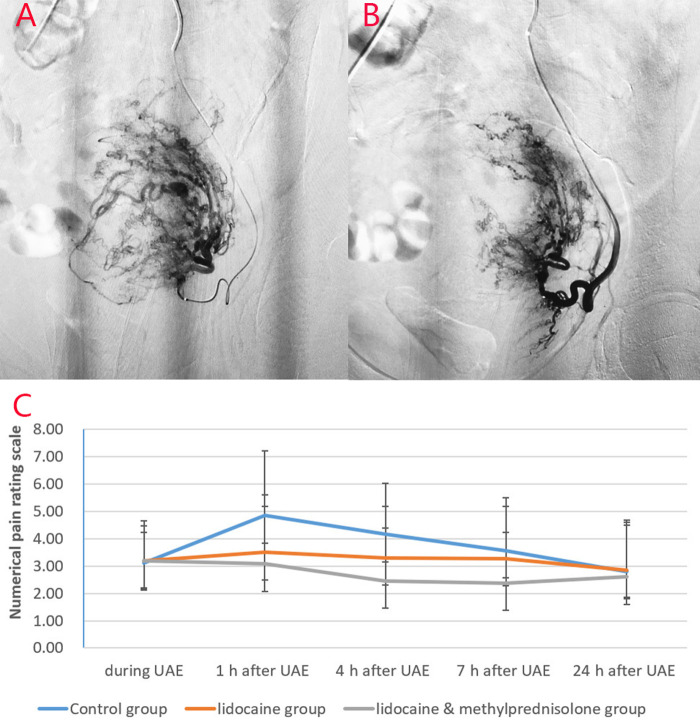
Images from a patient in the study group and a patient in the lidocaine group. (**A**) Before lidocaine injection and (**B**) after lidocaine injection. Left uterine arteriography images show uterine artery spasm after lidocaine injection, as indicated by the white arrow. (**C**) Pain score curve of the three groups.

Before embolization, uterine artery digital subtraction angiograms showed hypervascular uterine fibroids with dilated intramural arteries (**Figures 1B,C, 2A,B**). After UAE, the distal blood vessels were not evident, indicating successful embolization (**Figures 1E,F, 2D,E**). When embolic microspheres mixed with lidocaine were injected into the artery, obvious vasospasm was observed, resulting in an unclear development of the distal artery (**Figure 3A,B**).

### Evaluation of Pain Control and Dose of Sufentanil used During the First 24 h After UAE

The pain scores of the study group were significantly lower than those of the other two groups at 1, 4, and 7 h after embolization (*p* < 0.05) (**Table 4**). The pain score of the lidocaine group was significantly lower than that of the control group at 1 and 4 h after the operation. Moreover, the amounts of sufentanil used during the first 24 h after UAE in the three groups were significantly different. The amount of sufentanil used during the first 24 h in the study group was significantly lower than that in the control and lidocaine groups (*p* < 0.05) (**Table 4**). The pain score curve (**Figure 3C**) shows that the overall pain score trend of the lidocaine group was relatively gentle, and that of the study group was significantly lower in the first 7 h for pain control.

**Table 4:** Comparison of pain scores at different times and 24-hour sufentanil use among the three groups in patients with uterine artery embolism.

Numerical pain rating scale	During operation	1 h after UAE	4 h after UAE	7 h after UAE	24 h after UAE	Mean 24 h sufentanil dose(µg)
Control group (group 1)	3.11 ± 1.13	4.84 ± 2.36	4.16 ± 1.87	3.56 ± 1.93	2.80 ± 1.79	45.7 ± 6.51
Lidocaine group (group 2)	3.19 ± 1.47	3.50 ± 2.10	3.30 ± 1.88	3.28 ± 1.89	2.86 ± 1.82	38.3 ± 6.25
Study group (group 3)	3.18 ± 1.28	3.08 ± 2.09	2.46 ± 1.93	2.38 ± 1.85	2.60 ± 1.90	31.4 ± 4.16
All group						
F value or χ^2^ value	0.07	8.20	9.48	5.02	0.28	76.11
*P* value	0.94	0.01	0.01	0.01	0.78	0.01
Group 1 vs group 2						
Mean difference (95% CI)	−0.08 (−0.65–0.49)	1.62 (0.65–2.59)	0.85 (0.01–1.69)	0.28 (−0.56–1.11)	−0.06 (−0.87–0.75)	7.33 (4.83–9.83)
*P* value	0.77	0.01	0.04	0.51	0.88	0.01
Group 1 vs group 3						
Mean difference (95% CI)	−0.09 (−0.61–0.43)	1.76 (0.87–2.66)	1.70 (0.92–2.47)	1.17 (0.41–1.94)	0.20 (−0.55–0.95)	14.32 (12.02–16.62)
*P* value	0.73	0.01	0.01	0.01	0.59	0.01
Group 2 vs group 3						
Mean difference (95% CI)	−0.01 (−0.56–0.55)	0.14 (−0.81–1.09)	0.85 (0.03–1.67)	0.89 (0.08–1.71)	0.26 (−0.53–1.05)	6.99 (4.54–9.43)
*P* value	0.98	0.77	0.04	0.03	0.52	0.01

*Note: UAE, uterine artery embolization.*

## Discussion

This study confirmed that adding lidocaine to embolic microspheres during UAE resulted in pain control that was more effective than that observed in the control group, especially 4 h postoperatively, which is consistent with the results of previous studies (18). The optimal time for pain control during UAE is within 4 h, which may be related to the short half-life of lidocaine, which is 90–120 min (19). In the study group, intravenous infusion of methylprednisolone within 1 h after UAE provided an additional effect. Compared with the pain scores observed in the other two groups (control and study), addition of methylprednisolone increased postoperative pain control, reduced the use of opioids, and prolonged the analgesia time. However, the use of embolization microspheres mixed with lidocaine during embolization may cause uterine artery spasm, resulting in incomplete embolization. Moreover, if lidocaine is perfused before embolization, moderate to severe vasospasm will occur in the uterine artery, disrupting the therapeutic effect after embolization. Consequently, use of lidocaine for intrauterine artery perfusion is not allowed before embolization (20).

PES is caused by avascular necrosis of the muscle layer after UAE, which induces the release of cytokines and causes systemic inflammation (21). Previous studies have shown that glucocorticoids can reduce pain, nausea, and vomiting (22). The effect of methylprednisolone may be related to the anti-inflammatory effect and reduction in bradykinin (BK) levels by glucocorticoids (23). BKs are peptides resulting from proteolytic cleavage of high-molecular-weight kininogen produced by tissue injury, hypoxia, and inflammation. BKs serve as pain mediators that chemoattract neutrophils and inflammatory factors and participate in the process of inflammatory pain (24). Additionally, BKs can induce the release of inflammatory cytokines such as IL-6 and IL-8 through the NF-kB signaling pathway (25). Methylprednisolone produces significant anti-inflammatory effects by inhibiting the NF-kB pathway and upregulating anti-inflammatory mediators (13). Glucocorticoids can inhibit the vascular and cellular phases of inflammation and release of BKs, and reduce the inflammatory response secondary to ischemic changes (26). However, the use of glucocorticoids can cause elevation of white blood cell levels, and the greater the dosage, the more obvious the increase in leukocyte levels the following day (27). In this study, the difference in inflammation indicators between the three groups was not obvious because hormones also increased the white blood cell count. Therefore, the anti-inflammatory effect of methylprednisolone was not reflected by routine blood tests.

Sufentanil is a µ-opioid receptor agonist with a longer lasting effect than morphine and causes fewer adverse reactions (28). It is a safe and effective drug for controlling acute pain after embolization (28). Opioids may cause adverse reactions such as nausea and vomiting, with increased severity of adverse reactions directly related to the amount of opioids used (29). However, differences in the amounts of drugs administered to patients are related to individual clinical characteristics, surgery duration, and tumor type and size (30). Consequently, it is necessary to reduce the amount of sufentanil administered as much as possible to avoid the occurrence of related adverse reactions. Previous studies have shown that intra-arterial lidocaine can significantly improve pain after UAE and reduce the use of opioids (31). This study found that intravenous infusion of methylprednisolone after UAE significantly reduced the amount of sufentanil needed by patients after 24 h and was more effective than administration of lidocaine only.

The limitations of this study include its retrospective nature, limited sample size, single data source, and subjectivity of the pain score questionnaire. Dynamic changes in the inflammatory indices after embolization were not monitored, and the influence of glucocorticoids on postoperative pain within 24 h could not be determined. However, the relationship between the inflammatory indices within 24 h and postoperative pain scores could be compared to explore the specific role of inflammation after embolization.

## Conclusion

Increasing glucocorticoids to control postoperative pain in UAE patients is recommended as it will reduce the use of sufentanil and improve the postoperative experience and satisfaction of patients.

## Data Availability

The raw data supporting the conclusions of this article will be made available by the authors, without undue reservation.
